# *EGLN1* (PHD2) role in tumor microenvironment: insights for therapeutic targeting

**DOI:** 10.1038/s12276-025-01602-1

**Published:** 2025-12-19

**Authors:** Giulio Verna, Valentina Fantini, Alessandra Grieco, Alessia Ciarrocchi, Valentina Sancisi

**Affiliations:** 1 Laboratory of Translational Research, Azienda USL-IRCCS di Reggio Emilia, Reggio Emilia, Italy; 2 Clinical and Experimental Medicine PhD Program, University of Modena and Reggio Emilia, Modena, Italy

**Keywords:** Cancer microenvironment, Drug development

## Abstract

The tumor microenvironment (TME) is often hypoxic. *EGLN1*, which encodes the oxygen sensor PHD2, plays a crucial role not only in the survival of cancer cells but also in regulating other cell types that reside in the TME. In this Review, we explore the role of this protein in some of the key components of the TME, focusing on the functions of *EGLN1*/PHD2 in endothelial, stromal and immune cells. So far, the activity of *EGLN1*/PHD2 has been characterized in different cell types, albeit with controversial outcomes in different cancer settings. This Review aims to discuss the role of *EGLN1*/PHD2 in the TME and the strategies targeting this protein that might be used to hit tumors.

## PHD2 role in tumor microenvironment hypoxia explored

Oxygen is crucial for the survival of organisms, but low oxygen levels, or hypoxia, can occur in both normal and disease conditions. This study explores how cells respond to hypoxia, focusing on a protein called PHD2, which helps regulate another protein, HIF-1α, involved in this response. The authors discuss how PHD2 affects cancer growth and the tumor microenvironment, which includes surrounding cells and blood vessels. Research highlights that PHD2 plays a key role in controlling blood vessel formation and immune cell activity in tumors. By studying mice and cancer models, the authors found that reducing PHD2 activity can normalize blood vessels and improve oxygen delivery to tumors. This can enhance the effectiveness of cancer treatments by improving drug delivery and reducing metastasis. Researchers conclude that targeting PHD2 could be a promising strategy for cancer therapy.

This summary was initially drafted using artificial intelligence, then revised and fact-checked by the author.

## Introduction

Oxygen is an essential element for the survival and growth of all aerobic organisms. However, hypoxic conditions may be experienced in pluricellular organisms during normal life and in pathological conditions. In aerobic cells, hypoxia may have deleterious effects that are prevented by the activation of the hypoxia response pathway.

This pathway is triggered by the oxygen sensors, the prolyl-4-hydroxylase domain proteins (PHD1, PHD2 and PHD3, encoded by the *EGLN2*, *EGLN1*, and *EGLN3* genes, respectively), and the factor-inhibiting HIF (FIH). The PHD proteins hydroxylate proline residues (Pro402 and Pro564) on the HIF-α subunit in an oxygen-, iron- and α-ketoglutarate- (2-oxoglutarate, 2-OG) dependent manner. HIF-α hydroxylation leads to recognition by the von Hippel-Lindau tumor suppressor (pVHL), which targets HIF-α for proteasome degradation. Conversely, FIH hydroxylates the asparagine 803, leading to decreased affinity with co-activator p300/CBP^1–3^.

**Fig. 1:**
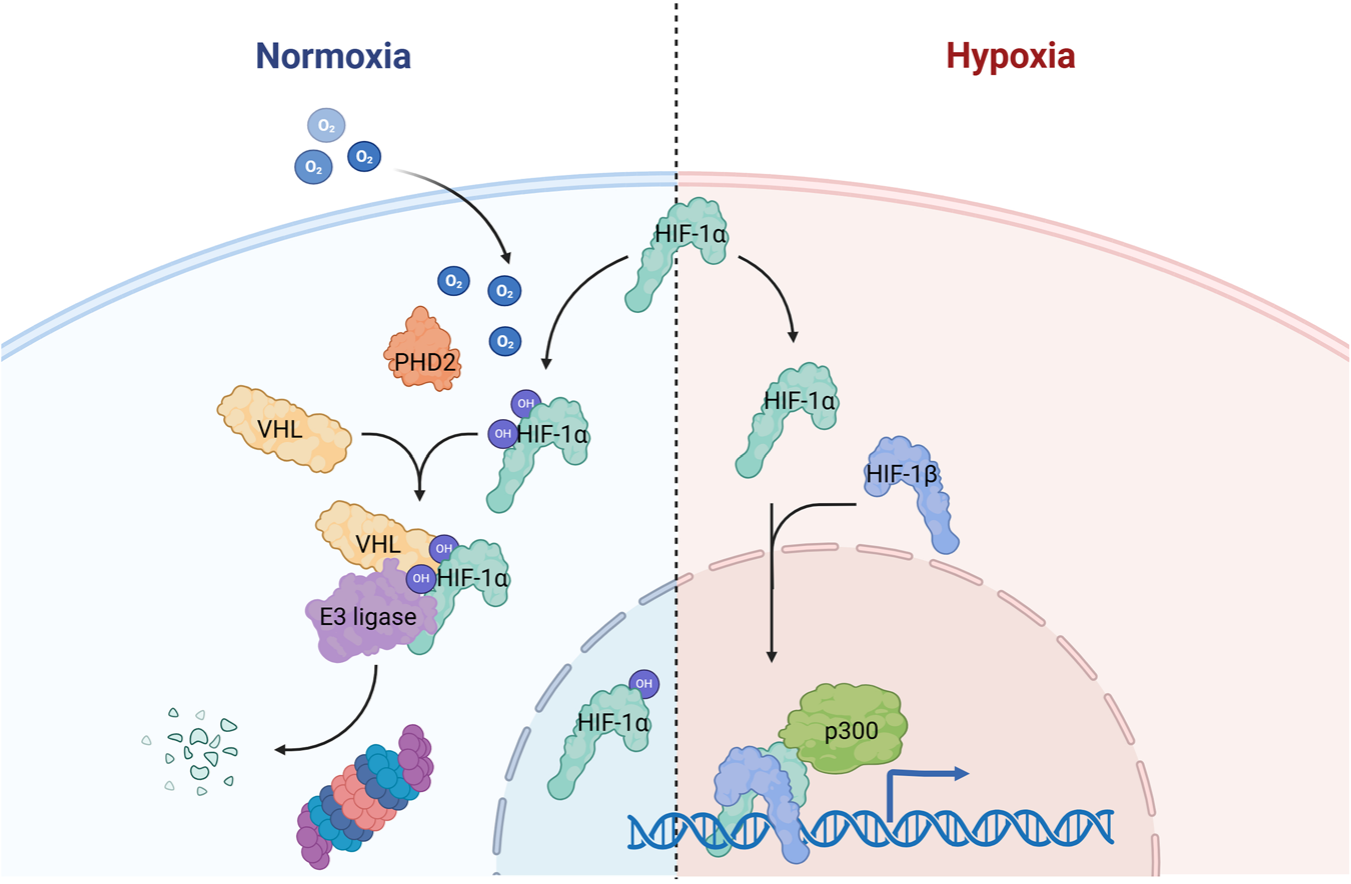
Hypoxia response pathway. In normoxia, the PHD enzyme hydroxylates the HIF-α subunit, leading to recognition by pVHL and degradation by the proteasome. In hypoxia, PHD enzyme is inactive and the HIF-α subunit is stabilized, enters the nucleus, dimerizes with HIF-β subunit and activates the transcription of hypoxic response genes. Created in https://BioRender.com.

The HIF-α subunit is encoded by three genes, *HIF1A*, *HIF2A* or *HIF3A*, and, together with the HIF-β subunit, constitutes the heterodimeric HIF transcription factor. In hypoxic conditions, the PHD hydroxylases are inactive, and the HIF-α subunit is stabilized and translocated into the nucleus, where it dimerizes with HIF-β and binds to hypoxia response elements on the regulatory regions of genes that need to be transcribed in response to hypoxia^1–3^ (Fig. 1).

*EGLN1*, encoding the PHD2 isoform, is the most abundant and ubiquitously expressed of the three *EGLN*s. Besides being dependent on oxygen, iron and 2-OG, several regulatory mechanisms converge on PHD2, finely tuning the cellular and systemic response to hypoxia^3^. Hereafter, *EGLN1* will be used to refer to the gene and PHD2 to the protein.

PHD2 is the preferential regulator of HIF-1α, playing a pivotal role during embryonic development and adult life in physiological conditions, as demonstrated by the embryonic lethality of its knockout in mice^4^.

In cancer, hypoxia and activation of hypoxia response pathways deeply influence both the tumor cells and the tumor microenvironment (TME). PHD2, being the primary oxygen sensor and the main regulator of HIF-1α, has been shown to influence the physiology of different TME cell populations^5^. In this Review, after discussing the complex relationship between PHD2/HIF and hypoxia in cancer cells, we will focus on the pivotal role of PHD2 in the response to hypoxia in TME cells. Finally, we will report pharmacological targeting strategies of the hypoxia pathway for cancer treatment and discuss open issues in including PHD2 inhibitors among these strategies.

## Hypoxia, *HIF* and *EGLN1* in cancer cells: a complex relationship

Hypoxia is highly prevalent in tumors owing to the rapid outgrowth of cancer cells, leading to structural and functional abnormalities in microvessels and increased diffusion distances. Importantly, hypoxia is widely associated with a worse prognosis in different cancer types^6^.

HIF-1α and HIF-2α have been frequently found aberrantly overexpressed in many different tumor tissues, compared with healthy counterparts, directly linking tumor hypoxia to HIF factor expression^7^. Meanwhile, different mechanisms besides hypoxia have been shown to induce HIF expression in cancer. In addition, the consequences of HIF activation may be variable as well, depending on HIF isoform and cancer type.

The inactivation of classical tumor suppressors, as well as many pro-oncogenic signals, leads to HIF activation. Loss of pVHL tumor suppressor, which is found in a familial angiomatous syndrome and the majority of sporadic cases of hemangioblastoma and clear-cell renal cell carcinoma (ccRCC), is the typical example that results in HIF-1α subunit stabilization due to lack of Cul2 ubiquitylation complex recognition^8^. However, the loss of PTEN and p53 has also been shown to promote HIF activity, with p53 directly contributing to HIF-1α degradation^9,10^. Several oncogenes, including *H-RAS*, *v-src* and *c-Myc*, have been shown to induce activation of HIF and its downstream target genes^11–13^. Many growth factors and intracellular signaling pathways activated during carcinogenesis can also stabilize and activate HIF-1α under normoxic conditions (for example, EGF, FGF2, insulin, IGF1-2, TNF, PDGF, AR, PI3K/AKT and BCR/ABL)^8,14–20^. HIF-1α subunits can be phosphorylated by different kinases on different threonine and serine residues with consequent stabilization or destabilization of the protein independently of oxygen tension^15,21–26^. Furthermore, other mechanisms, such as the regulation of mRNA stability and translation rate, may influence HIF levels in cancer cells^27,28^. Importantly, Rohwer and coworkers reported a poor association between intratumor hypoxic areas and HIF stabilization in colorectal cancer models, supporting a role for noncanonical HIF activation in promoting tumorigenesis^29^.

Although HIF-1α and HIF-2α have been widely shown to be overexpressed in many cancer types, they often play nonoverlapping and sometimes contrasting roles in cancer development and progression^30^. In most cases, HIF-1α seems to have a tumor-suppressive role, supported by its capacity to induce metabolic reprogramming, growth arrest and apoptosis, whereas HIF-2α is considered to have pro-oncogenic roles in inducing growth, pluripotency, and angiogenesis^30^. These differential functions are supported by distinct transcriptional programs regulated by the two factors^31^. However, the specific function of each factor (pro-oncogenic or tumor suppressor) is largely dependent on tumor type and, in some cases, also on tumor context. For example, in ccRCC, the respective pro-oncogenic role and tumor-suppressive role of HIF-1α and HIF-2α are well established^32,33^, but the recent development of a mouse model for this disease has questioned this concept. In this mouse model bearing an inducible deletion of *VHL*, *TP53* and *RB1* in renal cells, the HIF-1α deletion abrogated tumor growth, whereas HIF-2α had a limited effect. These results suggest a context-dependent effect of the two factors and a possible complementarity of their functions in cancer development^34^. Meanwhile, the transcriptional targets of the two factors also show a certain overlap, raising the question of whether they can compensate for each other^31^. Adding complexity to this field, HIF-1α factor expression has been frequently observed in the cytosolic compartment, and nontranscriptional roles of both HIF-1α and HIF-2α are emerging^35–37^.

The genes of the *EGLN* family have been reported to play variable roles in different cancer contexts. *EGLN3* is a tumor suppressor in most cases, whereas *EGLN1* and *EGLN2* have more controversial roles. *EGLN1*, in particular, has been described as a tumor suppressor in colorectal and pancreatic cancer^38,39^, as a pro-oncogenic factor in acute myeloid leukemia and in lung and ovarian carcinoma^40–42^, and as having dual roles depending on the study in breast and hepatocellular carcinomas^43–46^. These contradictory roles can be partly explained by the differential enzymatic activity of the PHD proteins on the two main HIF isoforms, with HIF-1α preferentially hydroxylated by PHD2 and HIF-2α by PHD1 and PHD3^5,47,48^. Nevertheless, a growing number of alternative PHD targets are being reported, including several proteins having a pivotal role in cancer development and progression, such as p53, AKT1, BRD4 and DYRK1^49–53^. In addition, all three PHD proteins have been shown to have still poorly characterized enzymatic-independent activities^54–56^. Together, this evidence suggests a widespread function of PHD proteins in sensing oxygen and other nutrient levels that go beyond their canonical role in HIF regulation.

PHDs belong to a wider family of 2-oxoglutarate-dependent dioxygenase (2-OGDD), which comprises approximately 60–70 members, including collagen hydroxylases, Jumonji C (JmjC) domain-containing demethylases, the ten-eleven translocation (TET) DNA demethylases and various RNA demethylases^57^. Although all these enzymes are dependent on oxygen for their activity, in many cases, it is unclear whether they may play the role of oxygen sensors, due to very high or unknown affinity for oxygen. Interestingly, KDM5A and KDM6A histone demethylases, belonging to the JmjC subfamily, can respond to oxygen level variations and modify histone methylation in a HIF-independent manner, leading to chromatin remodeling and gene expression regulation^58,59^.

Finally, the cells may sense oxygen also through other mechanisms. For example, Deygas and collaborators demonstrated that epithelial cells migrate toward areas of higher oxygen concentration in a HIF- and PHD-independent manner, mediated by EGFR’s ability to sense reactive oxygen species^60^.

**Fig. 2:**
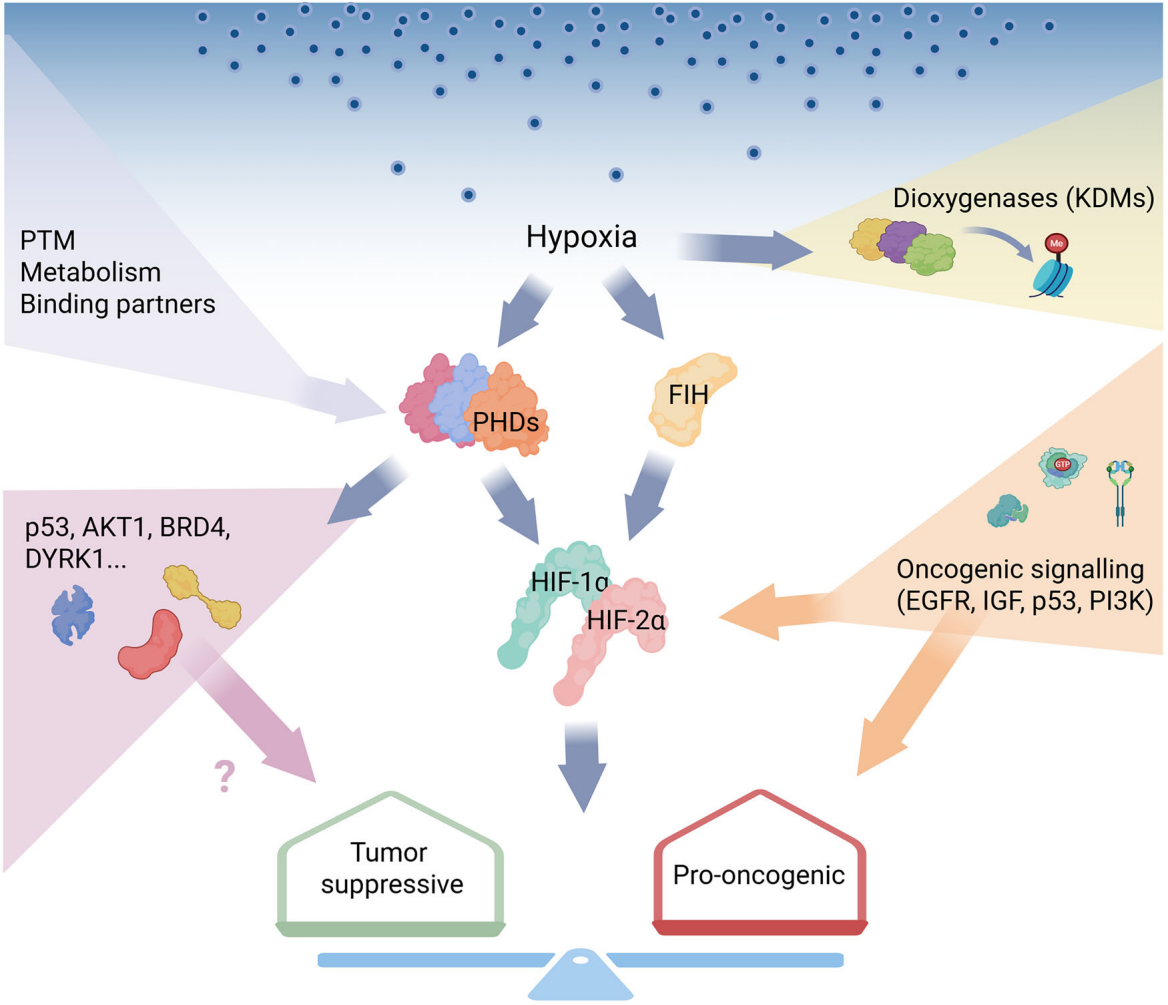
The multifaceted relationship among hypoxia, PHD enzymes and HIF factors in supporting or restraining tumor growth. Hypoxia can regulate other enzymes of the 2-OGDD family, besides the PHDs and FIH. Different mechanisms can regulate the activity of the PHD enzymes, including the presence of binding partners, the metabolic status of the cell and specific post-translational modifications (PTM). The HIF proteins are the main targets of PHDs, but several alternative targets have been described, including p53, AKT1, BRD4 and DYRK1, holding the possibility to influence tumor behaviour. Finally, a number of pro-oncogenic signals have been shown to stabilize the HIF-α subunit in a oxygen-independent way. Created in https://BioRender.com.

Taken together, these findings depict a complex regulatory network in which HIF factors are stabilized not only by hypoxia but also by other mechanisms, PHD enzymes have targets beyond HIFs, and the PHD–HIF axis is not the sole cellular oxygen sensor (Fig. 2). This complexity should be taken into consideration when studying hypoxia, both in cancer cells and in the TME.

## *EGLN1*-dependent response to hypoxia in the TME

Hypoxia affects cancer cells as well as host cell populations residing in the TME, including endothelial cells, fibroblasts and different immune populations. The *EGLN1* gene has been shown to have a key role in regulating the response to hypoxia in these populations, supporting various pro-oncogenic or tumor-suppressive functions.

### Angiogenesis

**Fig. 3:**
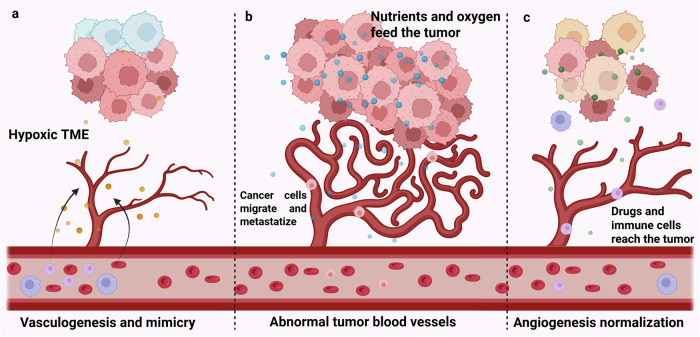
The relationship between hypoxia and angiogenesis in cancer. **a**,**b** The hypoxic TME shapes blood vessels and induces vasculogenesis (**a**) to feed the tumor and potentially lead to metastasis (**b**). **c** Angiogenesis normalization may lead to hypoxia relief, anticancer drug delivery and immune surveillance reactivation. Created in https://BioRender.com.

Vasculogenesis is classically defined as the de novo blood vessel formation from endothelial progenitor cells during embryonic life, whereas angiogenesis arises from the systemic and local need for nutrition and oxygen supply during adult life. Abnormal vessel formation is often associated with the onset of malignant disorders^61,62^. However, normal blood vessels distribute oxygen and nutrients evenly, whereas tumor blood vessels have an immature structure and high permeability^63^. These abnormal blood vessels mainly derive from tumor-induced angiogenesis, but alternative mechanisms may be involved, such as adult vasculogenesis and vascular mimicry exerted by tumor cells^64^. Increasing tumor volume, hypoxia and downstream activation of the HIF pathway induce angiogenesis to increase blood supply to cancer cells^65^. VEGF, the key soluble factor involved both in angiogenesis and vascular mimicry, is one of the most widely recognized targets of HIF transcription factors^66^. Based on these findings, antiangiogenetic drugs were developed and applied for cancer treatment in different settings. However, in most cancers, blood vessels are twisted, deregulated and dysfunctional^67–69^, indicating that vessel normalization might be the best strategy^70^. Indeed, blocking VEGF expression prunes immature vessels and increases their maturation^71^ (Fig. 3).

PHD2 appears to be the key PHD isoform regulating adult angiogenesis in mice, as its deficiency leads to hyperactive angiogenesis, whereas PHD1- or PHD3-deficient mice do not exhibit angiogenic defects^69^.

Moreover, in a syngeneic model of cancer growth, *EGLN1* haplodeficiency in host cells induces the normalization of the endothelial cell lining, leading to improved tumor oxygenation and drug diffusion, and to decreased metastatization^72,73^. In addition, inducible PHD2 KO in myeloid cells improves angiogenesis by attenuating the pro-inflammatory phenotype of macrophages^74^.

Complete PHD2 ablation in mouse endothelial and hematopoietic cells leads to extensive pulmonary vascular remodeling, including vascular occlusion and plexiform-like lesions, recapitulating the pathology of pulmonary arterial hypertension^75^. Conversely, PHD2 overexpression in endothelial cells suppresses hypoxia-induced cell proliferation^76^.

PHD2 ablation may enhance both angiogenesis and vasculogenesis, through increased HIF-1α stabilization as well as other collateral pathways. Indeed, PHD2 inhibition induces the release of pro-angiogenic factors, such as IL-8 and angiogenin, in a HIF-independent and NF-kB-dependent manner. These factors mediate tumor angiogenesis and the recruitment of bone marrow-derived cells^77^.

Overall, all the evidence indicates that PHD2 levels are fundamental for correct angiogenesis in physiological conditions. Conversely, in cancer, where blood vessel formation is aberrantly stimulated, decreasing PHD2 levels or activity may lead to angiogenesis normalization.

### Fibroblasts

Cancer-associated fibroblasts (CAFs) are directly or indirectly reprogrammed by cancer cells to be activated and produce extracellular matrix to favor microenvironment remodeling^78^ in several cancer types^79^, including pancreatic adenocarcinoma (PDAC)^80^, gastrointestinal cancer^81^ and breast cancer^79^.

Furthermore, the activated CAFs supply high-energy substances, including ketone bodies and lactate, to cancer cells through their autophagic pathways^82,83^.

The TME is highly oxidative. This activates two pro-autophagic pathways in stromal fibroblasts: one driven by HIF-1α and another orchestrated by NFκB^84^. As a result, CAFs initiate autophagy and mitophagy, leading to metabolic and proteomic reprogramming^85^. Autophagy sustains cancer cell growth because CAF debris and nutrients can be recycled. At the same time, HIF-1α-induced mitophagy enhances aerobic glycolysis, and CAFs secrete high-energy nutrients that can further boost oxidative metabolism in cancer cells^86^.

Nevertheless, autophagy in fibroblasts can also inhibit tumor progression in the early stages because the secreted intermediates might activate immune cells. Conversely, CAF autophagy promotes TME inflammation, hypoxia and the regulation of immune checkpoints at later time points in tumor development^87^.

In PDAC, myofibroblastic CAFs are characterized by αSMA expression and are considered to have tumor-restraining function, while inflammatory CAFs (iCAF) express lower levels of αSMA, produce inflammatory cytokines and enhance tumor growth^88^. Hypoxia potentiates the effects of cytokines secreted by PDAC cells, leading to a switch toward an iCAF phenotype, which is associated with worse cancer prognosis and therapy resistance. In this setting, HIF-1α stabilization induces the iCAF phenotype, while the secreted cytokines cooperate to boost HIF-1α transcriptional activity in a self-loop manner. This is mainly supported by IL-1α produced by PDAC cells in hypoxic conditions^89^.

However, prolonged hypoxia deactivates CAFs and reduces their ability to remodel and invade their surrounding matrix^90^. Indeed, in vitro treatment with PHD inhibitor dimethyloxalylglycine (DMOG) or PHD2 silencing leads to CAF deactivation and extracellular matrix remodeling in a HIF-1α-dependent manner. These findings are mirrored by results in orthotopic cancer models in which (DMOG) treatment or cotransplant with PHD2-silenced CAFs led to a marked decrease of metastatic load, without altering primary tumor growth^90^. Another report showed similar results in a spontaneous model of breast tumorigenesis, generated in the *EGLN1* haplodeficient background. These mice showed the same primary tumor growth as *EGLN1* wild type, but decreased metastases due to reduced CAFs activation and improved blood vessels. Moreover, the authors showed that CAF impairment was not due to PHD2 inhibition in CAFs, but rather in cancer cells. In fact, *EGLN1* haplodeficient cancer cells secrete less TGF-β, which in turn leads to reduced CAF activation^91^.

**Fig. 4:**
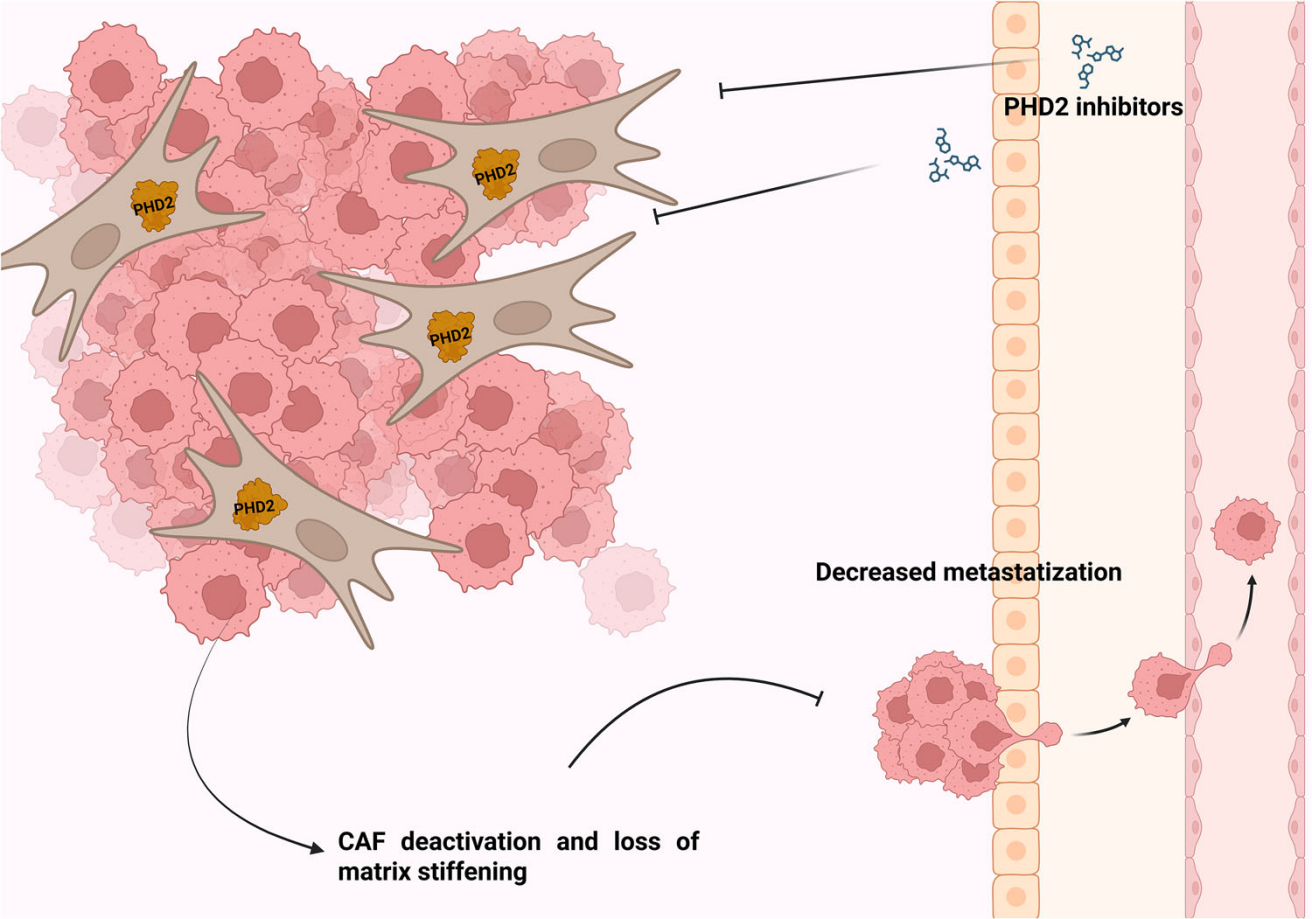
Effect of PHD inhibitors in CAFs. PHD2 inhibition in CAFs induces their deactivation, resulting in TME matrix remodeling and reduced metastatic potential of cancer cells. Created in https://BioRender.com.

Thus, inhibiting or reducing PHD2 activity might be crucial to inhibit tumor progression and CAF protumoral support. (Fig. 4).

### Macrophages

Tumor-associated macrophages (TAMs) can be clustered and characterized on the basis of their receptor expression, effector function and cytokine production. Several microenvironmental signals can induce M1 or M2 polarization in vivo and in vitro. Classically activated M1 macrophages are usually the effector cells that kill microorganisms and tumor cells, while alternatively activated M2 cells dampen inflammatory responses, scavenge debris and promote angiogenesis^92^. M1 macrophages secrete IL-12 and tumor necrosis factor (TNF), while M2 macrophages typically produce IL-10^93^.

M1-like macrophages can be found in the TME during the early phase of tumorigenesis when a pro-inflammatory response is still ongoing. They further activate effector immune cells, such as CD8^+^ cytotoxic T cells and natural killer cells^94,95^. TNF is a positive regulator of M1 polarization and a negative regulator of M2 polarization when the NF-κB pathway is activated. Furthermore, myeloid differentiation primary response 88 (MyD88) suppresses M2-associated gene expression in TAMs, resulting in an antitumor M1 phenotype polarization^96^.

An elevated M1/M2 TAM ratio is associated with a better prognosis in non-small cell lung cancer^97,98^, colorectal cancer^99^, ovarian cancer^100^, breast cancer^101^ and oral squamous cell carcinoma^102^. High M2 percentages, conversely, are linked to worse outcomes and thrive in a less inflammatory TME, favoring tumor growth^103^.

Strong hypoxic TME and large numbers of TAMs correlate with a decreased survival rate^104^. M2 macrophages express low levels of the MHCII complex or HLA-DR (in mice and humans, respectively) and were found in the most hypoxic areas inside human tumors^105^. Metabolism is tightly linked to TAMs functionality, in particular, M1, with high MHCII expression exhibiting a hampered TCA cycle. Conversely, M2 TAMs showed higher oxidative and glycolytic metabolism compared with M1 TAMs, which show less mitochondrial activity. These M2 TAMs can fuel their activity with lactate, increasing L-arginine metabolism and enhancing their T cell suppressive capacity^106^. HIF-1α is essential for macrophages activation and infiltration in vivo. *HIF1A* knockout in these cells results in impaired glycolytic capacity and drastic reduction of ATP production^107^. HIF-1α expression is also required for myeloid cells to kill pathogenic bacteria^107,108^.

M2 TAMs are associated with hypoxic regions, whereas M1 TAMs are located preferentially in better-oxygenated areas. *EGLN1* haplodeficiency induces vasculature normalization and enhances tumor oxygenation in mice. In this system, oxygen availability does not alter the attraction of monocytes to the tumor site nor the efficiency of monocyte differentiation into M2 or M1 TAMs. Less hypoxia does not influence M2 markers expression, albeit it downregulates the expression of typical genes involved in angiogenesis, metastasis and response to hypoxia. Laoui and colleagues concluded that tumor-infiltrating monocytes do not fail to differentiate because of hypoxia, but are instead influenced by other microenvironmental stimuli. They are attracted to hypoxic areas where they perform their protumoral activity^109^. In vitro, *EGLN1* knockout induces metabolic reprogramming of myeloid cells toward anaerobic glycolysis through increasing pyruvate dehydrogenase kinase 1 (PDK1) protein levels and decreasing pyruvate dehydrogenase enzyme activity, while *HIF1A* knockout inhibits this glycolytic reprogramming^110^. The inhibition of the glycolytic shift also suppresses macrophage migration and functionality^111^. These findings are in line with another report showing that depletion of HIF-1α in myeloid cells dramatically reduces glucose transporters and glycolytic enzyme expressions, resulting in a defective inflammatory response^110^.

**Fig. 5:**
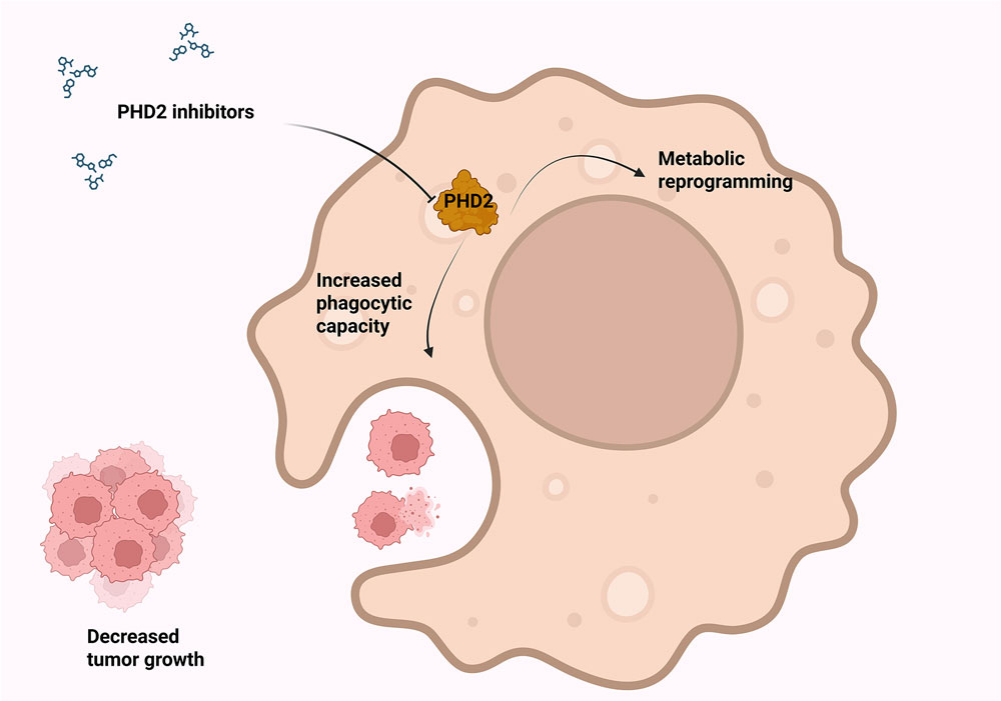
The effects of PHD2 inactivation on macrophage function in cancer TME. PHD2 genetic or pharmacological inhibition leads to metabolic reprogramming and increased phagocytic activity in TAMs. Created in https://BioRender.com.

Treatment with PHD2 inhibitor roxadustat in a syngeneic model of lung cancer and melanoma reduces tumor growth by increasing the macrophages’ phagocytic activity and by inducing tumor vessel normalization, through the PHD–HIF axis^112^ (Fig. 5).

### T lymphocytes

T cells are one of the most represented immune populations infiltrating solid tumors, being recruited to tumor sites by TME-secreted factors^113^. There, they can have different phenotypic differentiation and functions. Cytotoxic CD8^+^ T cells recognize tumor cells’ neo-antigens and kill tumor cells, creating perforin and granzyme pores on their membrane and inducing cell apoptosis through the IFNγ and TNF pathways^114^. According to their antitumoral function, their numbers are generally associated with a better prognosis^115^.

CD4^+^ T cells have more variable roles. In most cases, the anti-inflammatory TME primes them with regulatory functions (T_reg_). These T_reg_ cells have been characterized in several solid cancers. One of their main activities is to secrete anti-inflammatory cytokines, which hinder other T cell functions and promote tumor growth and metastasis^116^. CD4^+^ T cells may also differentiate into T helper cells, recruiting and activating natural killer cells and CD8^+^ T cells to the tumor site^117^.

The role of HIF-1α in T cells is rather controversial. Hypoxia induces a metabolic shift in CD8^+^ T cells, which enhances their antitumor activity^118^. On the other hand, hypoxic CD8^+^ T cells have decreased proliferative capacity due to metabolic remodeling^119^.

Nevertheless, the concomitant deletion of *EGLN1* and *EGLN3* genes in already primed CD8^+^ T cells enhanced their effector activity. These cells reduce tumor growth when adoptively transferred into several mouse cancer models. It is also relevant that culturing CD8^+^ T cells under hypoxic conditions boosted their antitumor activity in vivo through increased glucose metabolism and granzyme/perforin granule production^120,121^. This effect was independent of IFNγ secretion, even though its mRNA was upregulated.

Genetic deletion of *EGLN1* in T lymphocytes increases EG7-OVA tumor-killing activity. These cells have increased intracellular positive staining for IFNγ, TNF and granzyme B compared with wild-type controls^122^. The stabilization of HIF-1α in CD4^+^ T cells improves the effect of anti-PD-1 therapy to reduce tumor growth and patient survival. Similarly, VHL deletion in T cells, which leads to HIF-1α accumulation, prevents exhaustion in CD8^+^ T cells during chronic viral infections. Moreover, PHD protein expression in T cells is needed to induce tolerance against lung metastases due to an increase in T_reg_ cells and a decrease in CD8^+^ T cells’ effector function. Consequently, T cell-specific deletion of all three PHDs or pharmacological inhibition restrains lung colonization and improves the effect of adoptive transfer immunotherapy^123^.

**Fig. 6:**
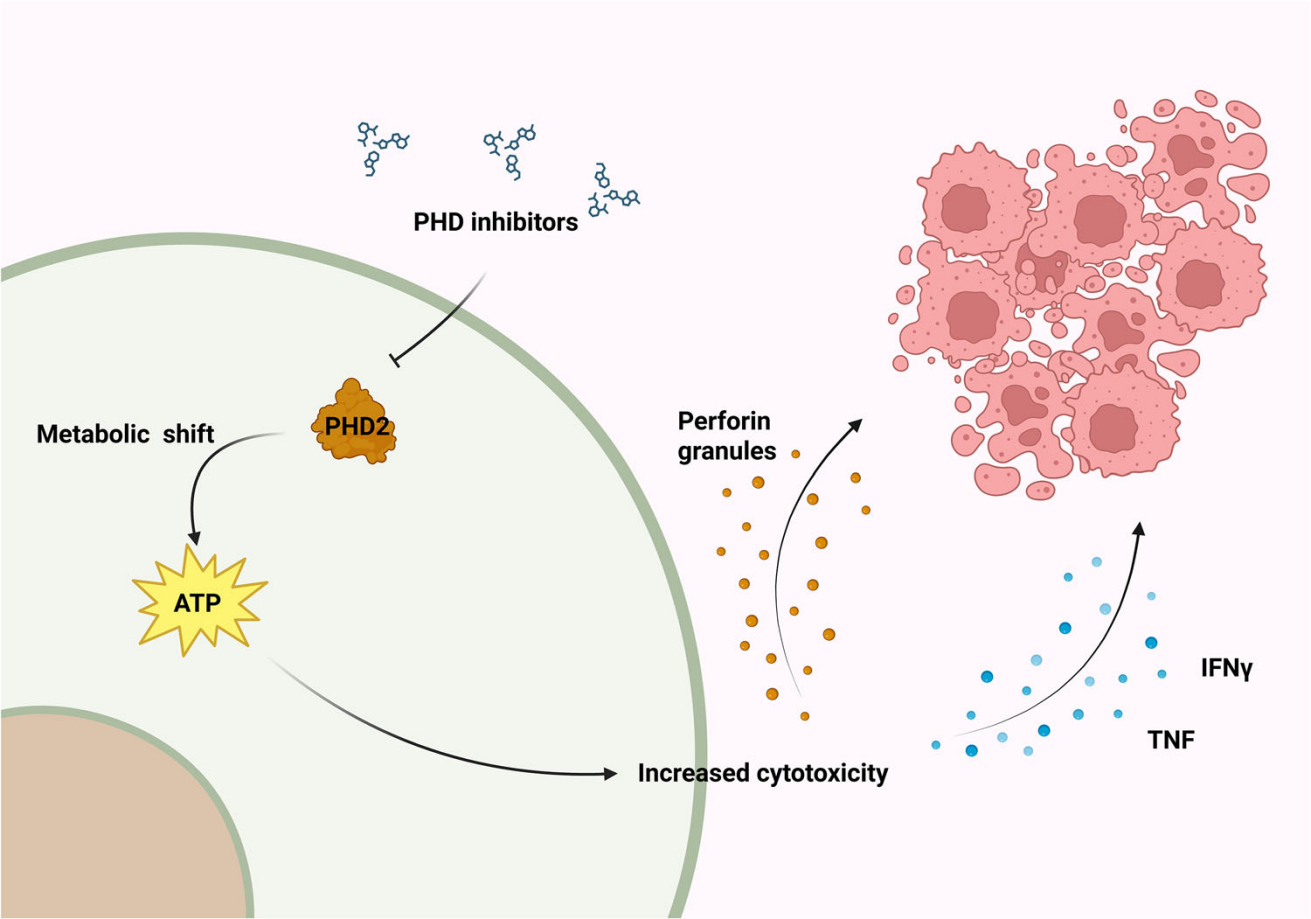
Effects of PHD2 inhibition in T lymphocytes. PHD2 inactivation shifts CD8^+^ T cell metabolism, resulting in an increased tumor killing potential. Created in https://BioRender.com.

Notably, PHD inhibitors activate CD8^+^ T cells through HIF-1α stabilization and induction of co-stimulatory molecules, leading to increased antitumor efficacy of adoptive T cell therapy^124^ (Fig. 6).

## Hypoxia pathway pharmacological targeting for cancer treatment: time to include PHD inhibitors?

**Fig. 7:**
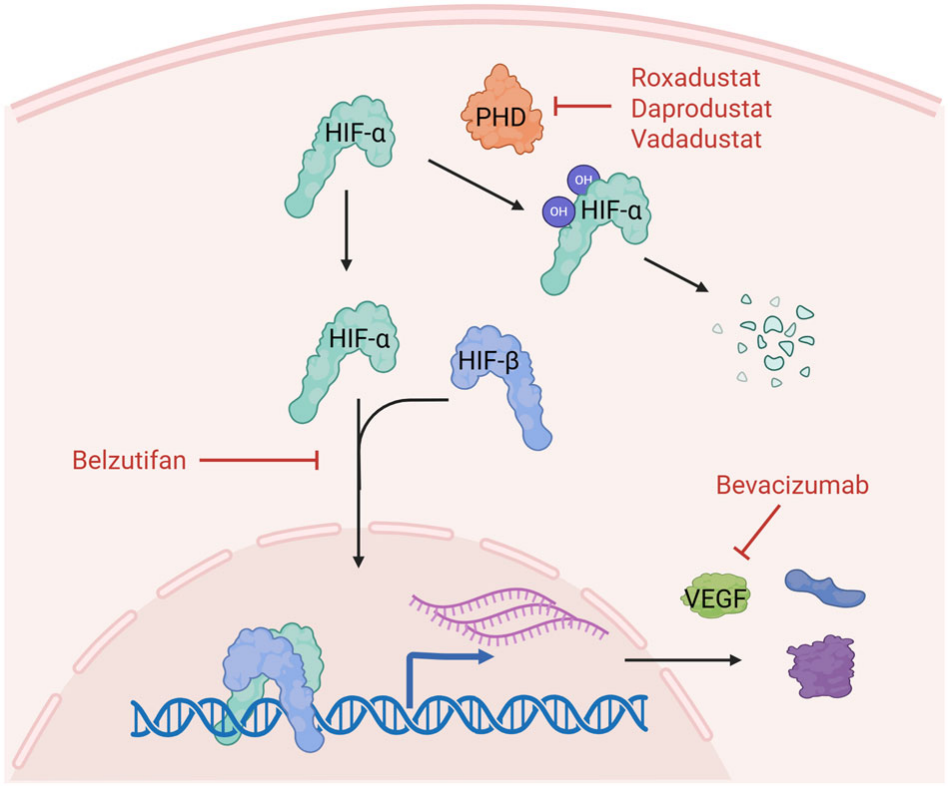
Pharmacological approaches to the hypoxia pathway. Several PHD enzymes inhibitors such as roxadustat, daprodustat and vadadustat have been developed for the treatment of anemia. Belzutifan is a small molecule specifically inhibiting the dimerization of HIF-2α with HIF-β. Inhibitors of the HIF transcriptional target VEGF have been used for more than 20 years for cancer treatment. Created in https://BioRender.com.

Due to the intimate association between hypoxia and tumors, targeting this pathway is regarded as a promising strategy to overcome different types of cancer. Major pharmacological targets in this pathway are the PHD enzymes, the HIF transcription factors and the HIF main effectors, such as VEGF (Fig. 7 and Supplementary Table 1). The latter is a well-established drug target, with specific monoclonal antibodies such as bevacizumab that have been in clinical practice for almost 20 years. Anti-VEGF drugs were originally developed to inhibit hypoxia-induced angiogenesis, aiming to starve tumors and restrain metastatic spreading. However, the efficacy of these treatments was limited in monotherapy and remains circumscribed to some cancer types in combination with other agents. Notably, other mechanisms of action emerged, such as vasculature normalization and immune modulation, partially explaining tumor-specific effects and indicating the possibility of more rational combinations^125^.

Due to the pivotal role of HIF transcription factors in cancer cell regulation and their multifaceted role in promoting an immune-suppressive microenvironment, they have been considered promising targets for a long time. Several agents have been shown to directly or indirectly target HIF factor expression, protein synthesis, stabilization and dimerization. However, most of these inhibitors regulate HIFs indirectly and/or in addition to other pathways, making it difficult to discriminate the HIF-dependent effects^30,126^. In 2009, Scheuermann and collaborators discovered a cavity within the PAS-B domain of HIF-2α, which is not present in HIF-1α, leading to the development of specific inhibitors of HIF-2α dimerization with HIF-β subunit^127^. Belzutifan is the most advanced of these inhibitors and demonstrated efficacy in preclinical models and clinical trials of VHL-related cancers such as ccRCC and hemangioblastoma, receiving US Food and Drug Administration approval for these indications in 2021^128,129^. Combinations of belzutifan with other anticancer agents, including immunotherapy, are currently under investigation in several clinical trials^130^.

A relatively recent strategy proposes to alleviate TME hypoxia itself, resulting in simultaneous downregulation of HIF activity and TME reprogramming, including the promotion of immune cell recruitment and activation. These effects may be achieved by vascular normalization, leading to increased oxygen and nutrient concentration but also fostering immune cell infiltration and anticancer drug delivery^131,132^.

Mazzone and colleagues developed a syngeneic mouse model carrying *EGLN1* haplodeficiency in the host animal and showed that PHD2 deficiency leads to vessel normalization, improving tumor perfusion and oxygenation, and restraining metastatic spreading^72^. In this model, loss of PHD2 also results in better chemotherapy delivery and protection of normal organs from chemotherapy side effects^133^. In addition, *EGLN1* deletion in T cells promotes the differentiation of effector T cells, improving tumor control and response to immune checkpoint blockade treatment^122^. Notably, PHD2 genetic or pharmacologic inhibition in cancer cells has variable effects depending on cancer type, showing anti-oncogenic activity in melanoma, lung, ovarian and osteosarcoma cancer^40,41,134^. Taken together, this evidence indicates that PHD2 systemic inhibition through pharmacologic approaches may exert anticancer activity through a double effect on cancer cells and TME, at least in some cancer types.

PHD enzyme inhibitors have been developed for the treatment of other diseases, such as anemia and ischemia^135^. Clinical trials of different PHD inhibitors showed efficacy in the management of anemia associated with chronic kidney disease with limited side effects, leading to the approval of roxadustat, daprodustat and vadadustat for the treatment of this pathology in different countries^136^. These results indicate that the use of PHD inhibitors may also be safe in neoplastic disease and can be repurposed as agents capable of influencing both the cancer cells and the TME.

Notably, all the available inhibitors rely on competition with the 2-OG cofactor, which is shared by all three enzymes and also by the other dioxygenases of the superfamily. As the three PHD enzymes play different roles in different cancer contexts, specificity issues may arise. All the tested inhibitors induce erythropoiesis in vivo, while they show some isoform specificity in vitro^135^. However, due to differential functions of the PHD proteins in cancer, the activity of each inhibitor on the three PHD enzymes and downstream pathways should be carefully evaluated before repurposing in the oncology context. Furthermore, the activity of these inhibitors on other dioxygenases is often poorly investigated and should be taken into consideration. Ideally, the development of isoform-selective inhibitors would be optimal for the precise targeting of these enzymes and for limiting undesired side effects.

## Conclusions

In summary, *EGLN1*/PHD2 is known as a key regulator in the cellular response to hypoxia. Furthermore, it can trigger a plethora of other metabolic and regulatory mechanisms that affect the TME as a whole. PHD2 activities can have different outcomes based on the context, including the tumor subtype or the TME cell population. In most cases, however, PHD2 triggers a cascade of events that promote tumor growth, drive abnormal blood vessel formation, and suppress immune cell activity. For these reasons, inhibiting PHD2 may represent a novel and effective anticancer strategy, as it affects both cancer cells and the TME. Although several PHD inhibitors are already in clinical practice for the treatment of anemia, specificity issues may arise and limit their use in oncology. Therefore, the generation of new, stronger and more selective inhibitors may be beneficial for the optimal targeting of this intriguing protein for anticancer therapy.

## Supplementary information

Supplementary Information
