# Supplementary material for: EGLN1 (PHD2) role in tumor microenvironment: insights for therapeutic targeting
Source: Exp Mol Med. 2025 Dec 19;57(12):2739–48. doi: 10.1038/s12276-025-01602-1 (PMC12800280; doi:10.1038/s12276-025-01602-1)
Supplement: Supplementary file 1 — Supplementary Information [file 12276_2025_1602_MOESM1_ESM.pdf]

Supplementary Table 1. Drugs targeting the hypoxia pathway

Drug	Target	Mechanism	Approval	Reference
**PHD inhibitors**				
Roxadustat	PHDs	Competition with 2-OG cofactor binding	EMA	Chen 2019 [1]
Daprodustat	PHDs		FDA	Singh 2022 [2]
Vadadustat	PHDs		EMA	Eckardt 2021 [3]
**HIF inhibitors**				
Belzutifan	HIF2	Inhibits dimerization	EMA/FDA	Choueiri 2024 [4]
Panobinostat	HDAC	Decrease HIF protein/activity	EMA/FDA	Verheul 2008 [5]
Vorinostat	HDAC		FDA	Zhang 2017 [6]
Digoxin	HIF1/2	Inhibits HIF translation	EMA/FDA	Zhang 2008 [7]
Apigenin	PI3K	Block the PI3K/AKT/mTOR pathway, which inhibits HIF1a synthesis and activation	**-**	Fang 2005 [8]
Everolimus	mTOR		EMA/FDA	Majumder 2004 [9]
Bortezomib	Proteasome		FDA/EMA	Befani 2012 [10]
Topotecan	Topoisomerase inhibitor	Inhibits HIF translation	FDA/EMA	Rapisarda 2004 [11]
PX-478	HIF1/2	Decreases HIF transcript and protein level	-	Koh 2008 [12]
LW6	VHL	Induces VHL expression, promoting HIF degradation	-	Lee 2010 [13]
**VEGF inhibitors**				
Bevacizumab	VEGF	Monoclonal antibody against VEGF	EMA/FDA	Garcia 2020 [14]

1. Chen, N. *et al.* Roxadustat Treatment for Anemia in Patients Undergoing Long-Term Dialysis. *N Engl J Med* **381**, 1011–1022 (2019).
2. Singh, A. K. *et al.* Efficacy and Safety of Daprodustat for Treatment of Anemia of Chronic Kidney Disease in Incident Dialysis Patients: A Randomized Clinical Trial. *JAMA Intern Med* **182**, 592–602 (2022).
3. Eckardt, K.-U. *et al.* Safety and Efficacy of Vadadustat for Anemia in Patients Undergoing Dialysis. *N Engl J Med* **384**, 1601–1612 (2021).
4. Choueiri, T. K. *et al.* Belzutifan versus Everolimus for Advanced Renal-Cell Carcinoma. *N Engl J Med* **391**, 710–721 (2024).
5. Verheul, H. M. W. *et al.* Combination strategy targeting the hypoxia inducible factor-1 alpha with mammalian target of rapamycin and histone deacetylase inhibitors. *Clin Cancer Res* **14**, 3589–3597 (2008).

6. Zhang, C. *et al.* Vorinostat suppresses hypoxia signaling by modulating nuclear translocation of hypoxia inducible factor 1 alpha. *Oncotarget* **8**, 56110–56125 (2017).
7. Zhang, H. *et al.* Digoxin and other cardiac glycosides inhibit HIF-1alpha synthesis and block tumor growth. *Proc Natl Acad Sci U S A* **105**, 19579–19586 (2008).
8. Fang, J. *et al.* Apigenin inhibits VEGF and HIF-1 expression via PI3K/AKT/p70S6K1 and HDM2/p53 pathways. *FASEB J* **19**, 342–353 (2005).
9. Majumder, P. K. *et al.* mTOR inhibition reverses Akt-dependent prostate intraepithelial neoplasia through regulation of apoptotic and HIF-1-dependent pathways. *Nat Med* **10**, 594–601 (2004).
10. Befani, C. D. *et al.* Bortezomib represses HIF-1α protein expression and nuclear accumulation by inhibiting both PI3K/Akt/TOR and MAPK pathways in prostate cancer cells. *J Mol Med (Berl)* **90**, 45–54 (2012).
11. Rapisarda, A. *et al.* Topoisomerase I-mediated inhibition of hypoxia-inducible factor 1: mechanism and therapeutic implications. *Cancer Res* **64**, 1475–1482 (2004).
12. Koh, M. Y. *et al.* Molecular mechanisms for the activity of PX-478, an antitumor inhibitor of the hypoxia-inducible factor-1alpha. *Mol Cancer Ther* **7**, 90–100 (2008).
13. Lee, K. *et al.* LW6, a novel HIF-1 inhibitor, promotes proteasomal degradation of HIF-1alpha via upregulation of VHL in a colon cancer cell line. *Biochem Pharmacol* **80**, 982–989 (2010).
14. Garcia, J. *et al.* Bevacizumab (Avastin®) in cancer treatment: A review of 15 years of clinical experience and future outlook. *Cancer Treat Rev* **86**, 102017 (2020).
